# The diversity between curatively resected pancreatic head and body-tail cancers based on the 8th edition of AJCC staging system: a multicenter cohort study

**DOI:** 10.1186/s12885-019-6178-z

**Published:** 2019-10-22

**Authors:** Weiwei Sheng, Ming Dong, Guosen Wang, Xiaoyang Shi, Wei Gao, Kewei Wang, He Song, Gang Shi, Xiaodong Tan

**Affiliations:** 1 Department of gastrointestinal surgery, the First Hospital, China Medical University, Shenyang, 110001 China; 2 Department of general surgery, Cancer hospital of China Medical University, Shenyang, 110042 China; 3 Department of thyroid and pancreatic surgery, Shengjing Hospital of China Medical University, Shenyang, 110004 China

**Keywords:** Pancreatic head and body-tail cancers, Clinical significance, Prognosis, 7th and 8th edition of AJCC staging system

## Abstract

**Background:**

To our knowledge, there are no studies to systematically compare the detailed clinical significance between curatively resected pancreatic head (ph) and body-tail (pbt) ductal adenocarcinoma based on the new 8th edition of AJCC staging system (8th AJCC stage) that was just applied in clinical practice in 2018.

**Methods:**

Three hundred fifty-one patients with curatively resected pancreatic adenocarcinoma (PC) from three center hospitals were entered into this multicenter cohort study.

**Results:**

Increasing tumor size (*P* < 0.001), T stage (T1 + T2 vs T3 + T4, *P* = 0.003), frequent postoperative liver metastasis (PLM) (*P* = 0.002) and 8th AJCC stage (IA to VI, *P* < 0.001; I + II vs III + IV, *P* = 0.002) were closely associated with the progression of pbt cancers compared with that in ph cancer patients. Moreover, tumor size≥3 cm (*P* = 0.012), 8th AJCC stage (III + IV) (*P* = 0.025) and PLM (*P* = 0.010) were identified as independent risk factors in pbt cancers in logistic analysis. Patients with pbt cancers had a significantly worse overall survival compared with ph cancer patients (*P* = 0.003). Moreover, pbt was an independent unfavorable factor in multivariate analysis (*P* = 0.011). In addition to lymph nodes metastasis, 8th AJCC stage, vascular invasion and PLM, increasing tumor size and advanced T stage were also closely associated with the poor prognosis in 131 cases of pbt cancer patients compared with Ph cancer patients.

**Conclusion:**

Pbt, as an independent unfavorable factor for the prognosis of PC patients, are much more aggressive than that in ph cancers according to 8th AJCC staging system. 8th AJCC staging system are more comprehensive and sensitive to reflect the malignant biology of pbt cancers.

## Background

From 2000 to 2011, pancreatic adenocarcinoma (PC) takes up the second upward trend of age-standardized mortality rates in the Chinese male population [1]. Meanwhile, it is the fourth most common cause of cancer death in the United States and Japan [2, 3]. Despite advances in multimodality treatment, long-term survival hasn’t shown improvement over the past several decades [4]. The poor prognosis of PC is mainly due to the late diagnosis and advanced progression, most patients with PC are diagnosed at stages III and IV [5]. Even following curative resection, the reported 5-year survival rate remains low (7–24%) [6]. Accurate evaluation of tumor stage is a prerequisite for further treatment and prognostic prediction. The AJCC TNM staging system has been widely applied worldwide as the most authorized tool for tumor staging assessment. AJCC released the 8th edition (8th AJCC stage), which incorporated significant changes in the T and N classification of PC [7].

Most studies in terms of PC focuse on the head of the pancreas (ph), whereas rare data is regarding pancreatic tail and body (pbt) cancers. Previous studies investigate the incidence rate and survival time between ph and pbt ductal cancers [8]. However, the results remain controversial and the relationship between tumor location and clinical characters is rarely reported. Meanwhile, to the best of our knowledge, there is no studies to systematically compare the clinical significance between curatively resected ph and pbt cancers based on the new8th AJCC stage [8]. Based on the new 8th AJCC stage, we find new clinical difference between curatively resected ph and pbt cancers, which provides a new clinical sight in revealing the malignant biology of PC, especially in pbt cancer.

## Methods

### Patients

**Fig. 1:**
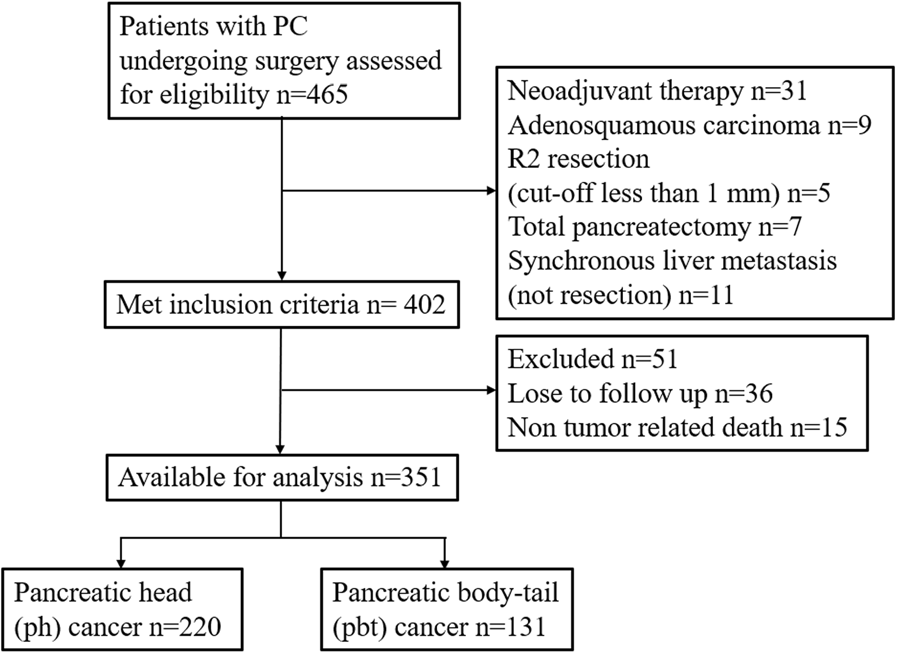
Study flow chart. Undergoing strict selection, 351 cases of PC patients were finally entered into this study from three multiple centers. PC: pancreatic adenocarcinoma; Ph: pancreatic head; Pbt: pancreatic body-tail

**Fig. 2:**
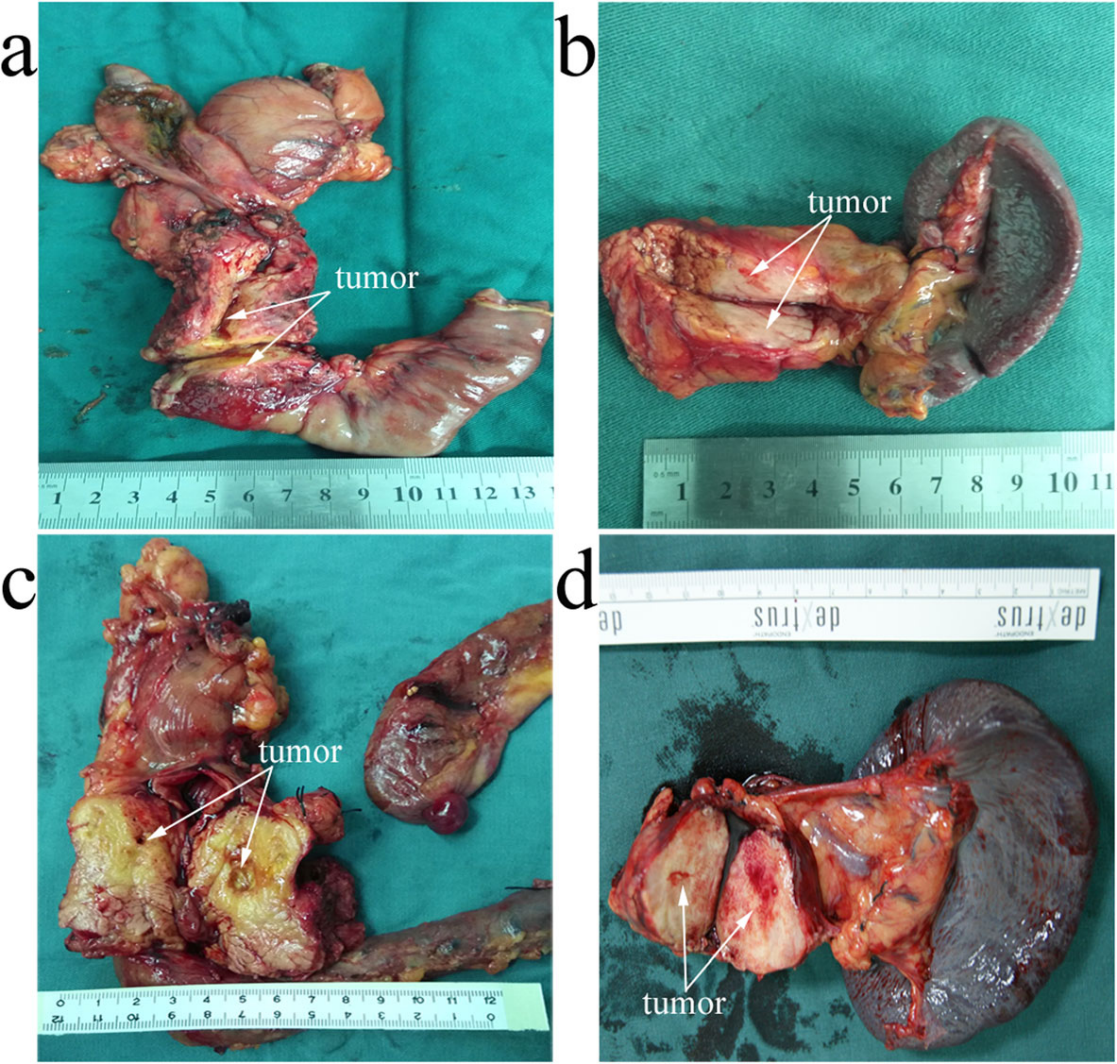
Four classic samples from consecutive PC patients underwent PD or PDP. **a, c** Under PD treatment, two ph tumor samples was shown as arrows suggested. **b, d** Under PDP treatment, two pbt tumor samples was shown as arrows suggested. PD: Pancreatoduodenectomy; PDP: distal pancreatectomy. Ph: pancreatic head; Pbt: pancreatic body-tail

This research protocol was approved by the ethical committee of the institutional review board of China Medical University and a consent form was signed by each participating patient. All patients enrolled from the First hospital of China Medical University, Shengjing hospital of China Medical University and Cancer hospital of China Medical University were histologically proven to be pancreatic ductal adenocarcinomas. Contrast computed tomography (CT)/positron emission tomography (PET), contrast nuclear magnetic resonance (MRI) and surgical exploration were used to ensure whether all PC patients meet our resection criteria as Sugiura et al. previously reported [9], including: a) no distant metastasis, b) tumor extension to the superior mesenteric artery or celiac trunk was less than 90°and can be completely resected and constructed. The detailed enrollment procedure was shown in Fig. 1. Based on above criteria, 351 cases of consecutive PC patients underwent pancreatoduodenectomy (PD) or distal pancreatectomy (PDP) were finally entered into this study between 2008 and 2016. In order to achieve R0 resection, cancer resection margins were at least 1 mm as cut-off. Meanwhile, some cases with peripancreatic invasion underwent corresponding organ resection, such as spleen, left adrenal gland, gastrointestine (partial stomach, duodenum, intestine or colon), artery (hepatic, superior mesenteric and celiac artery) and vein (portal or superior and inferior mesenteric vein). 6 PC patients were detected a single liver metastasis (preoperative CT examination is not detected) in surgery, we additionally executed partial hepatectomy. A dedicated table for patients’ characteristics was summarized in Table 1. Four classic samples from consecutive PC patients underwent radical PD and PDP showed in Fig. 2.

**Table 1:** The clinical data in 351 cases of PC patients with curatively surgical resection

Parameters	No. of patients	Parameters	No. of patients
Cases	351	Cases	351
Age (years)		Perineural invasion	
≤ 65	240	Absent	278
> 65	111	Present	73
Gender		Vascular permeation	
Male	210	Absent	266
Female	141	Present	85
Tumor size (cm)		Pre-therapeutic CA19–9 level	
< 3	108	Mean ± SD	355 ± 283
≥ 3	243	PLM	
Tumor location		Absent	227
Ph	220	Present	124
Pbt	131	7th AJCC stage^a^	
Differentiation		IA	19
Well	140	IB	140
Moderate	118	IIA	76
Poor	93	IIB	104
8th T stage		IV	6
T1	35	Surgical procedures	
T2	167	PD alone	196
T3	143	PD + gastrointestine	8
T4	6	PD + portal or superior mesenteric vein	11
7th T stage^a^		PD+ hepatic or superior mesenteric artery	3
T1	27	PD + liver	2
T2	215	PDP alone	97
T3	103	PDP + gastrointestine	12
Lymph nodes metastasis		PDP + portal vein or inferior mesenteric vein	6
N0	244	PDP + gastrointestine +left adrenal	5
N1	91	PDP + liver+ left adrenal	1
N2	16	PDP + liver	3
8th AJCC stage		PDP + left adrenal	4
IA	24	PDP+ celiac artery	3
IB	119	Postoperative chemotherapy	
IIA	93	Ph cancers	125/220
IIB	86	Pbt cancers	73/131
III	23		
IV	6		

*N1* Lymph nodes metastasis 1–3; *N2* Lymph nodes metastasis> 3; *PLM* postoperative live metastasis; *PD* Pancreatoduodenectomy; *PDP* Distal pancreatectomy*7th and 8th AJCC stage* 7th and 8th edition of AJCC staging system in PC; *Ph* Pancreatic head; *Pbt* Pancreatic body-tail. a 6 cases of T4 stage in 8th AJCC stage (III) were exclude in 7th AJCC stage

### Follow-up

All patients were followed up by the operating surgeons. As described previously [6], postoperative patients were performed routinely laboratory examinations, including tumor markers, liver function, US, abdominal CT/PET or contrast MRI every 3–6 months. For postoperative liver metastasis (PLM), if the liver metastasis showed no definite evidence of other metastasis or recurrence elsewhere, we characterized the newly developed hepatic lesion as PLM [10]. Patient follow-up examinations was performed each 3 months for the first 2 postoperative years, every 6 months for > 2 years, and yearly thereafter. One hundred twenty-five cases of ph cancer patients (125/220, 56.8%) and 73 cases of pbt cancer patients (73/131, 55.7%) accepted postoperative gemcitabine-based chemotherapy, no difference was shown in two groups with or without chemotherapy treatment.

### Statistical analysis

Statistical analysis was performed using SPSS software 19.0. The differences between curatively resected ph and pbt cancers was analyzed using a Chi-Squared test. A logistic regression analysis was performed to determine the pathologic impact findings that were significant with regard to differences in the univariate analysis. The Kaplan-Meier method was used to estimate PC patients’ survival, and differences were analyzed by the log-rank test. The variables that were significant by the univariate analysis were subjected to a multivariate Cox proportional hazards regression analysis in a stepwise manner. A value of *P* < 0.05 was considered to be statistically significant.

## Results

### Comparison of the 7th and 8th editions of the TNM staging system for patients

The detailed information of 7th and 8th AJCC stage in PC was summarized in Additional file 1: Table S1 and Additional file 2: Table S2. Briefly, in the 8th edition, stages T1-T3 are redefined according to tumor size. When the tumor invades the celiac axis, hepatic artery and/or superior mesenteric artery, it is defined as T4, and the classification as “unresectable” was removed. Because all the patients enrolled in this study accept the curative resection, 6 PC patients with T4 stage (III stage) based on 8th AJCC stage were exclude in 7th AJCC system (Table 1). In addition, the N classification was further subdivided according to the number of positive lymph nodes as N0, N1 and N2. T1–3N2M0 was defined as stage III in 8th AJCC stage, while it was defined as stage IIB in 7th AJCC stage. In current study, 14.9% (16/107) of these patients had metastasis more than 3 lymph nodes (pN2) (Table 1). The ratio of stage IA, IB, IIA, IIB, III and IV of 8th AJCC stage was 6.8, 33.9, 26.4, 24.3, 6.5 and 1.7%, respectively, while the ratio of stage IA, IB, IIA, IIB and IV was 5.5, 40.5, 22, 30.1 and 1.7% in 7th AJCC stage (III stage that was defined as “unresectable” PC were excluded).

### Different clinical significance between ph and pbt cancers in 351 cases PC patients with curative resection

Chi-Squared test in Table 2 showed that tumor size, T stage, 8th AJCC stage and PLM were significantly different between ph and pbt cancers. Increasing tumor size ≥3 cm (ph 60.7% vs 81.6%; *P* < 0.001), frequent PLM (ph 29% vs pbt 45.8%, *P* = 0.002) and advanced T (T3 + T4, ph 36.3% vs pbt 52.7%, *P* = 0.003) and 8th AJCC stage (IA to VI, *P* < 0.001; III + IV, ph 5.2% vs pbt 14.5%, *P* = 0.002) were closely associated with the progression of pbt cancers compared with that in ph cancers. However, age, gender, tumor differentiation, lymph nodes metastasis, CA199 level and perineural and vascular invasion showed no difference (*P* > 0.05). A multivariate analysis (logistic regression analysis) identified tumor size≥3 cm (*P* = 0.012), 8th AJCC stage (III + IV) (*P* = 0.025) and PLM (*P* = 0.010) as independent risk factors in pbt cancers (Table 2). It was worthy noted that T and TNM stage based on 7th AJCC stage system showed no significant difference in both cohorts, which implying a close relationship of pbt cancers with the advanced clinal stage based on 8th AJCC stage.

**Table 2:** Clinical significance between ph and pbt cancers in 351 cases PC patients with curatively resection

Parameters	No. of patients	Chi square		*P*	Multivariate analysis Odds ratio (95% CI)	*P*
		Head Body-tail				
Cases	351					
Age (years)						
≤ 65	240	157	83	0.125		
> 65	111	63	48			
Gender						
Male	210	131	79	0.911		
Female	141	89	52			
Tumor size (cm)						
< 2	35	28	7	0.027		
≥ 2	316	192	124			
Tumor size (cm)						
< 3	108	84	24	0.000	2.133(1.180–3.856)	0.012
≥ 3	243	136	107			
Differentiation						
Well	140	86	54	0.793		
Moderate	118	73	45			
poor	93	61	32			
Lymph nodes metastasis						
N0	244	155	89	0.101		
N1	91	59	32			
N2	16	6	10			
7th T stage^a^						
T1 + T2	242	159	83	0.114		
T3	103	58	45			
7th AJCC stage^b^				0.360		
IA	19	13	6			
IB	140	86	54			
IIA	76	45	31			
IIB	104	71	33			
IV	6	2	4			
8th T stage						
T1 + T2	202	140	62	0.003	1.344(0.805–2.243)	0.258
T3 + T4	149	80	69			
8th AJCC stage				0.000		
IA	24	18	6			
IB	119	84	35			
IIA	93	42	51			
IIB	86	66	20			
III	23	8	15			
IV	6	2	4			
8th AJCC stage						
I + II	321	209	112	0.002	2.520(1.121–5.665)	0.025
III + IV	30	11	19			
Perineural invasion						
Absent	278	180	98	0.118		
Present	73	40	33			
Vascular permeation						
Absent	266	172	94	0.174		
Present	85	48	37			
Pre-therapeutic CA19–9 level						
Mean ± SD	**/**	389.6 ± 255.7	324.2 ± 283.3	0.429		
PLM						
Absent	227	156	71	0.002	1.854(1.160–2.963)	0.010
Present	124	64	60			

*N1* Lymph nodes metastasis 1–3; *N2* Lymph nodes metastasis> 3; *PLM* postoperative live metastasis; *7th and 8th AJCC stage* 7th and 8th edition of AJCC staging system in PC; *Ph* Pancreatic head; *Pbt* Pancreatic body-tail. a, b 6 cases of T4 stage in 8th TNM stage (III) were exclude

### Prognostic factors of PC patients who underwent curative pancreatectomy

**Fig. 3:**
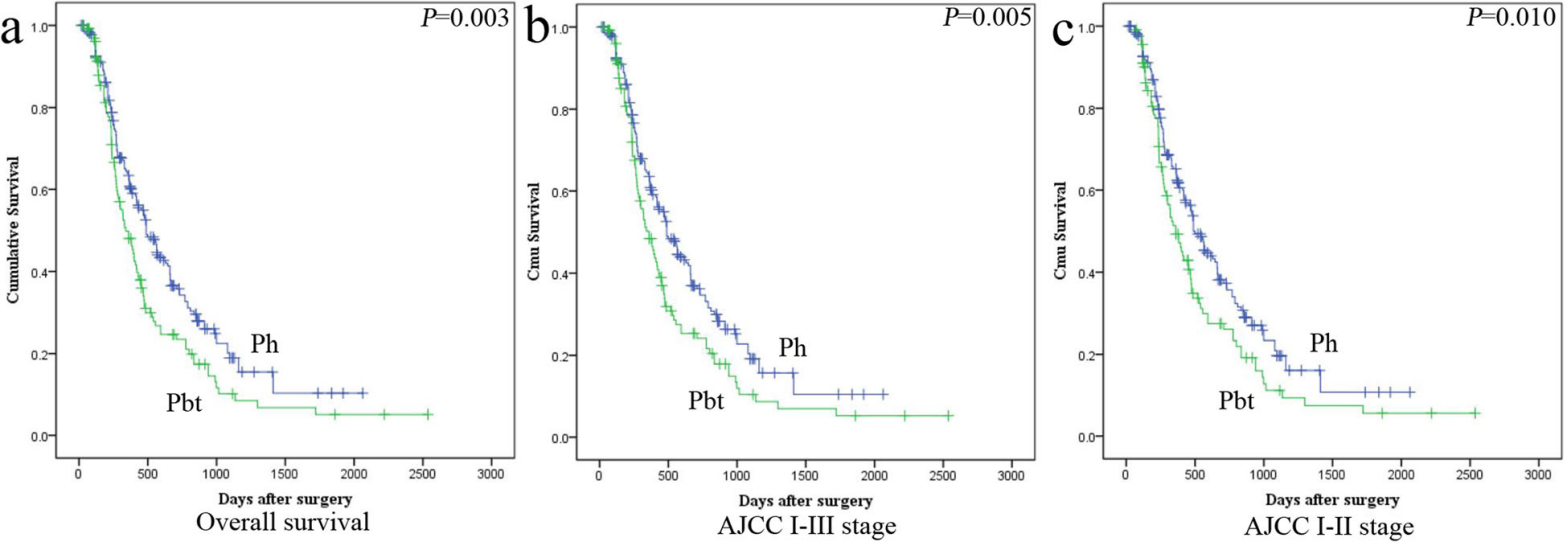
The prognosis between ph and pbt cancers with different clinical stage of 8th AJCC. **a.** The prognosis between ph and pbt cancers in 8th AJCC stage I to III. **b.** The prognosis between ph and pbt cancers in 8th AJCC stage I to III. **c.** The prognosis between ph and pbt cancers in 8th AJCC stage I to II

Patients with pbt cancers had a significantly worse overall survival compared with ph cancer patients (*P* = 0.003) in univariate analysis (Table 3) (Fig. 3a). Meanwhile, lymph nodes metastasis (*P* = 0.001), 8th AJCC stage (*P* = 0.007), vascular permeation (*P* = 0.004) and PLM (*P* < 0.001) were also associated with PC patients’ poor prognosis. In multivariate model, tumor location (*P* = 0.011), lymph nodes metastasis (*P* = 0.004), 8th AJCC stage (*P* = 0.012) and PLM (*P* = 0.001) were independent unfavorable prognostic indicators in PC (Table 3). 7th AJCC stage was also associated with the poor prognosis of PC patients (*P* = 0.012). Interestingly, previous studies show that pbt cancer patients have a better prognosis than ph cancer patients in early 7th AJCC I and II stage [11]. In current study, pbt cancer patients showed worse prognosis in both 8th AJCC I-III stage and I-II stage compared with ph cancer patients (Fig. 3b, c). Only in 8th AJCC I stage, the median survival days of pbt cancer patients was longer than that in ph cancer patients, but no statistic difference (data not shown). In addition, lymph node metastasis (N0/N1) in 7th AJCC stage failed to stratify patients by survival, whereas lymph node metastasis (N0/N1/N2) based on 8th AJCC stage was an independent unfavorable prognostic indicator in our current study. It indicated that lymph node metastasis in 8th AJCC stage is more comprehensive to reflect the malignant progression and poor prognosis of PC patients.

**Table 3:** Univariate and multivariate analysis for prognostic factors in 351 cases of PC patients with curatively surgical resection

Parameters	median survival (days)	Univariate analysis *P* (log rank)	Multivariate analysis hazard ratio (95% CI)	*P*
Age (< 65/ ≥65 years)	432/421	0.127	─	
Gender (male/female)	421/472	0.366	─	
Tumor location (ph/pbt)	488/340	0.003	1.405(1.082–1.826)	0.011
Tumor size (< 2/ ≥2 cm)	472/421	0.371	─	
Tumor size (< 3/ ≥3 cm)	472/418	0.096	─	
Well/Moderate/poor Differentiation	452/420/382	0.071	─	
T stage (T1 + T2/ T3 + T4)	472/381	0.068	─	
Lymph nodes metastasis 8th (N0/N1/N2)	480/330/284	0.001	1.451(1.123–1.874)	0.004
Lymph nodes metastasis 7th (N0/N1 + N2)^a^	472/399	0.090	─	
8th AJCC stage (I + II /III + VI)	468/284	0.007	1.442(1.085–1.915)	0.012
Perineural invasion (absent/present)	454/400	0.179	─	
Vascular permeation (absent/present)	480/330	0.004	1.401(0.905–2.168)	0.131
CA19–9 level (< 37 U/ml/ ≥37 U/ml)	565/395	0.104	─	
PLM (absent/present)	499/330	0.000	1.594(1.224–2.076)	0.001
7th AJCC stage	468/172	0.012	Not included	

*N1* Lymph nodes metastasis 1–3; *N2* Lymph nodes metastasis> 3; *7th and 8th AJCC stage* 7th and 8th edition of AJCC staging system in PC; *Ph* Pancreatic head; *Pbt* Pancreatic body-tail. a In 7th AJCC stage, N1 and N2 combined together

### Different prognostic indicators in ph and pbt cancer patients with curative surgical resection

Lymph node metastasis, 8th AJCC stage, vascular invasion and PLM were associated with the poor prognosis in 220 cases of Ph cancer patients (Table 4). In 131 cases of pbt cancer patients, in addition to above characters, tumor size and T stage were identified as the poor prognostic indicators (Table 4). More clinical factors based on 8th AJCC stage were the prognostic indicators in pbt cancer compared with the ph cancer.

**Table 4:** Difference of prognostic factors in Ph and Ptb cancer patients with curatively surgical resection

Tumor location	Parameters	Median survival (days)	Univariate analysis *P* (log rank)
220 Ph cancers	Age (< 65/ ≥65 years)	499/480	0.131
	Gender (male/female)	454/615	0.335
	Tumor size (< 3/ ≥3 cm)	555/488	0.358
	Well/Moderate/poor Differentiation	615/555/411	0.155
	T stage (T1 + T2/T3)	488/454	0.105
	Lymph nodes metastasis (N0/N1/N2)	565/418/185	0.004
	8th AJCC stage (I + II /III + IV)	360/273	0.017
	Perineural invasion (absent/present)	880/545	0.298
	Vascular permeation (absent/present)	565/350	0.005
	CA19–9 level (< 37 U/ml/ ≥37 U/ml)	666/450	0.171
	PLM		
	absent/present	586/365	0.039
131 Pbt cancers	Age (< 65/ ≥65 years)	381/300	0.111
	Gender (male/female)	395/340	0.439
	Tumor size (< 3/ ≥3 cm)	530/320	0.023
	Well/Moderate/poor Differentiation	400/280/273	0.070
	T stage (T1 + T2/ T3 + T4)	418/320	0.016
	Lymph nodes metastasis (N0/N1/N2)	360/259/234	0.007
	8th AJCC stage (I + II /III + IV)	499/273	0.001
	Perineural invasion (absent/present)	360/333	0.104
	Vascular permeation (absent/present)	400/265	0.009
	CA19–9 level (< 37 U/ml/ ≥37 U/ml)	468/331	0.099
	PLM	432/275	0.001
	absent/present		

*N1* Lymph nodes metastasis 1–3; *N2* Lymph nodes metastasis> 3; *8th AJCC stage* 8th edition of AJCC staging system in PC; *Ph* Pancreatic head; *Pbt* Pancreatic body-tail

## Discussion

8th AJCC stage demonstrates a more equal distribution among stages and increases prognostic accuracy compared with 7th AJCC stage. In an international multicenter cohort study including 1525 consecutive patients, the new T stage does not demonstrate significant correlation with survival on univariate or multivariate analysis, whereas the new N stage showed accurate discrimination of survival [12]. These results were consistent with our current study. However, the superiority of the 8th edition in evaluating the relationship between tumor location and clinical characters has not been investigated in PC patients, to our knowledge. Based on the new 8th AJCC stage, we found new diversity between ph and pbt cancers from a multicenter cohort study.

In anatomy, cell composition, blood supply, lymphatic and venous backflow,

and innervations are significantly different between ph and pbt cancers [13]. In clinic, tumors at different locations (ph vs pbt) display different clinical presentation,

treatment efficiency (surgery and chemoradiotherapy) and prognosis [14]. The incidence rate for ph cancer has remained at 5.6% per 100,000, whereas the rate for pbt cancers has increased by 46% between 1973 and 2002 in the SEER database [7]. Though both ph and pbt cancers had a higher proportion diagnosis in the distant stages (a neoplasm that has spread to parts of the body remotes from the primary tumor or to distant lymph nodes), patients with ph cancer were more likely to have localized and regional diseases (12.9 and 32.2%, respectively) as compared with pbt cancers (6.6 and 13.9%, respectively) [7]. According to 7th AJCC stage, there was no significant difference in TNM stage between resected ph and pbt cancers [15]. However, in current study, we find new clinical difference between curatively resected ph and pbt cancers bases on 8th AJCC stage, which hasn’t been reported previously to our knowledge.

The alteration of the definitions of T and N is the main changes in 8th AJCC stage compared with the 7th AJCC stage [16]. Just shown in Additional file 1: Table S1 and Additional file 2: Table S2, extra-pancreatic invasion can be difficult to predict accurately before surgery and may be inconsistently assessed by pathologists [17]. T3 tumors are now defined as those ≥4 cm, while nodal involvement has been improved from a binary system to one based on extent of nodal involvement. In current study, increasing tumor size and advanced T stage and 8th AJCC stage were closely associated with the progression of pbt cancers compared with ph cancers. Only one study shows tumor size but not T and clinical stage in 7th AJCC stage exhibits difference in resected ph (56 cases) and pbt (24 cases) cancers [15], which is consistent with our study. Based on the alteration of T and N status in 8th AJCC stage, T1–3 stage was likely a stratified analysis of tumor size. Meanwhile, new 8th AJCC stage mainly increased III stage (16 vs 0) but decreased IIB stage (86 vs 104) in PC patients compared with 7th AJCC stage in our study, which is the critical reason for the discrimination in above results just as Omar Abdel-Rahman suggested [18]. We additionally found PLM was more frequent in pbt cancers, which is consistent with the study by Maria Chiara Ambrosetti et al. [19]. However, Nakata B et al. show that the recurrence of peritoneum, liver, lung and bone showed no difference in tumor location [15]. Among 707 unresectable PC patients with stage III, 30.1% developed PLM. However, no risk factors were identified among these patients [20]. The inconsistence might be due to the different sample size and diversity in national population included in different studies.

Currently, prognostic difference between ph and pbt cancer patients remain controversial. Data from SEER database (1988–2004) including 33,752 PC patients presents a significant lower median survival (4 months vs 6 months) in patients with pbt cancer compared with those with ph cancer [21]. However, data from the national PC registry of Japan showed a significant lower 5-year survival rate (10.7% vs 13.8%) for patients with ph cancers (*n* = 5788) than those with pbt cancers (*n* = 1629) [22]. Both unresectable and resectable PC patients are enrolled in above studies. In our current study, we only enrolled curatively resected PC patients from three multiple centers. Our study showed that pbt cancer patients had a worse survival compared with ph cancers and was an independent unfavorable prognostic factor. However, a Japanese study enrolling. Eighty consecutive patients with resectable PC presents similar overall survival and recurrence rates after a curative resection between ph (*n* = 56) and pbt (*n* = 24) cancers [15]. Wentz SC et al. also show no relationship of tumor location (151 ph vs 18 pbt) with resected PC patients [23]. Interestingly, in 43,946 PC patients from SEER registry database, higher survival rates is shown in ph cancer compared with pbt cancer in several variables (age, sex, race, geography, and time). But the 3-year survival rate for local-stage (neoplasm confined to the organ of origin) pbt cancer is 20.0% compared with 9% for local-stage in ph cancer [7]. In 32 PC patients with 7th AJCC stage II, both overall and tumor-free survival were significantly higher in the patients with pbt cancer compared with those with ph cancers [11]. Our study showed that the survival time of pbt cancer patients was longer than that in ph cancer patients only in 8th AJCC I stage but no statistic difference. Indeed, some small metastases (liver metastasis) known as “micrometastases” from PC may be overlooked even with advanced imaging and surgical exploration [24]. In our study, 6 PC patients had a simultaneous single liver metastasis resection that was not detected in preoperative examination. 4 of 6 patients were evaluated in early stage (less than IIA) if we neglected the small single liver metastasis. Generally, pbt cancers were associated with much more advanced stage and worse prognosis in PC patients.

Finally, compared with ph cancers, we first showed tumor size and T stage were not only independent risk factors in the development of pbt cancers, but also poor prognostic indicators based on 8th AJCC stage. Taken together, 8th AJCC stage are more comprehensive to reflect the poor prognosis of pbt cancer patients.

### Limitations

Generally, one limitation in this study is that we don’t have a systematical standardization in surgical procedure and postoperative pathological examination throughout 3 centers, resulting in unstablebilty in lymph node yield, tumor size, and margin status [25, 26]. In addition, the sample size is still small in our current study. That is the reason that some important clinical characters, such as tumor differentiation (*P* = 0.071), only get bordering statistic association with PC patients’ survival. Finally, our study enrolls a few patients with extended R0 resection (combining with surrounding organ resection) in both cohorts that is recommended according to NCCN guidelines but might bring some confounder in current study. Two relatively larger studies show favorable results following hepatic metastasis resection for PC in a highly selected cohort of patients [27, 28]. That is one reason that we enroll 6 cases with synchronous hepatectomy for the single liver metastasis that was not found by pre-operative enhanced CT. Because only 2 and 4 cases of synchronous hepatectomy are included in ph and pbt cohorts, respectively, it has little effect in our statistic results even though we deleted these 6 cases.

## Conclusion

Based on the 8th AJCC staging system, tumor size, T stage, AJCC stage and PLM are independent risk factors in the development of pbt cancers compared with ph cancers. Pbt, as an independent unfavorable factor for the prognosis of PC patients, are much more aggressive than that in ph cancers according to 8th AJCC staging system. 8th AJCC staging system are more comprehensive and sensitive to reflect the malignant biology of pbt cancers compared with ph cancers.

## Supplementary information

**Additional file 1:** **Table S1.** 8th AJCC stage for PC. The details of TNM Stage in 8th edition of American Joint Committee on Cancer according to primary tumor, regional lymph node and Distant metastasis.

**Additional file 2:** **Table S2.** 7th AJCC stage for PC. 7th AJCC stage for PC. The details of TNM Stage in 7th edition of American Joint Committee on Cancer according to primary tumor, regional lymph node and Distant metastasis.

## Data Availability

The datasets used and/or analyzed during the current study are available. from the corresponding author on reasonable request.

## Abbreviations

7th AJCC stage: 7th edition of AJCC staging system
8th AJCC stage: 8th edition of AJCC staging system
Pbt: Pancreatic body-tail
PC: Pancreatic adenocarcinoma
PD: Pancreatoduodenectomy
PDP: Distal pancreatectomy
Ph: Pancreatic head
PLM: Postoperative liver metastasis

## References

[1] Chen W, Zheng R, Baade PD, Zhang S, Zeng H, Bray F (2016). Cancer statistics in China, 2015. CA Cancer J Clin.

[2] Siegel RL, Miller KD, Jemal A (2016). Cancer statistics. CA Cancer J Clin.

[3] Kanno A, Masamune A, Hanada K, Maguchi H, Shimizu Y (2017). Multicenter study of early pancreatic cancer in Japan. Pancreatology.

[4] Ryan DP, Hong TS, Bardeesy N (2014). Pancreatic adenocarcinoma. N Engl J Med.

[5] UICC (2017). TNM classification of malignant tumors.

[6] Sheng W, Dong M, Zhou J (2016). Yuji li, Fanmin Kong, Yulin tian. Tumor size and clinical stage are independent risk predictors for the high occurrence and poor prognosis of postoperative liver metastasis in patients with radically resectable pancreatic cancer. Int J Clin Exp Pathol.

[7] Shi S, Hua J, Liang C, Meng Q, Liang D, Xu J (2019). Proposed modification of the 8th edition of the AJCC staging system for pancreatic ductal adenocarcinoma. Ann Surg.

[8] Lau MK, Davila JA, Shaib YH (2010). Incidence and survival of pancreatic head and body and tail cancers: a population-based study in the United States. Pancreas.

[9] Sugiura T, Uesaka K, Mihara K, Sasaki K, Kanemoto H, Mizuno T (2013). Margin status, recurrence pattern, and prognosis after resection of pancreatic cancer. Surgery.

[10] Park JB, Kim YH, Kim J (2012). Radiofrequency ablation of liver metastasis in patients with locally controlled pancreatic ductal adenocarcinoma. J Vasc Interv Radiol.

[11] Ling Q, Xu X, Ye P, Xie H, Gao F, Hu Q (2017). The prognostic relevance of primary tumor location in patients undergoing resection for pancreatic ductal adenocarcinoma. Oncotarget.

[12] van Roessel S, Kasumova GG, Verheij J, Najarian RM, Maggino L, de Pastena M (2018). International validation of the eighth edition of the American joint committee on Cancer (AJCC) TNM staging system in patients with resected pancreatic Cancer. JAMA Surg.

[13] Ling Q, Xu X, Zheng SS, Kalthoff H (2013). The diversity between pancreatic head and body/tail cancers: clinical parameters and in vitro models. Hepatobiliary Pancreat Dis Int.

[14] Kikuyama M, Kamisawa T, Kuruma S, Chiba K, Kawaguchi S, Terada S (2018). Early Diagnosis to Improve the Poor Prognosis of Pancreatic Cancer. Cancers (Basel).

[15] Nakata B, Yamada N, Amano R, Tendo M, Inoue M, Sakurai K (2007). Comparison of clinicopathological characteristics of curatively resected pancreatic head and body/tail ductal cancers. J Exp Clin Cancer Res.

[16] Kamarajah SK, Burns WR, Frankel TL, Cho CS, Nathan H (2017). Validation of the American joint commission on Cancer (AJCC) 8th edition staging system for patients with pancreatic adenocarcinoma: a surveillance, epidemiology and end results (SEER). Ann Surg Oncol.

[17] Adsay NV, Bagci P, Tajiri T, Oliva I, Ohike N, Balci S (2012). Pathologic staging of pancreatic, ampullary, biliary, and gallbladder cancers: pitfalls and practical limitations of the current AJCC/UICC TNM staging system and opportunities for improvement. Semin Diagn Pathol.

[18] Abdel-Rahman O (2018). Evaluation of the 8th AJCC staging system for pathologically versus clinically staged pancreatic adenocarcinoma: a time to revisit a dogma?. Hepatobiliary Pancreat Dis Int..

[19] Ambrosetti MC, Zamboni GA, Mucelli RP (2016). Distribution of liver metastases based on the site of primary pancreatic carcinoma. Eur Radiol.

[20] D S, W L, GY B, YH F, SX H, MZ Q (2017). Risk factors of liver metastasis from advanced pancreatic adenocarcinoma: a large multicenter cohort study. World J Surg Oncol.

[21] Artinyan A, Soriano PA, Prendergast C, Low T, Ellenhorn JD, Kim J (2008). The anatomic location of pancreatic cancer is a prognostic factor for survival. HPB (Oxford).

[22] Matsuno S, Egawa S, Fukuyama S, Motoi F, Sunamura M, Isaji S (2004). Pancreatic Cancer registry in Japan: 20 years of experience. Pancreas.

[23] Wentz SC, Zhao ZG, Shyr Y, Shi CJ, Merchant NB, Washington K (2012). Lymph node ratio and preoperative CA 19-9 levels predict overall survival and recurrence-free survival in patients with resected pancreatic adenocarcinoma. World J Gastrointest Oncol.

[24] Hatwell C, Zappa M, Wagner M, Michoux N, Paradis V, Vilgrain V, Maggiori L, Panis Y (2014). Detection of liver micrometastases from colorectal origin by perfusion CT in a rat model. Hepatobiliary Pancreat Dis Int.

[25] Chandrasegaram MD, Goldstein D, Simes J (2015). Meta-analysis of radical resection rates and margin assessment in pancreatic cancer. Br J Surg.

[26] Soer E, Brosens L, van de Vijver M (2018). Dilemmas for the pathologist in the oncologic assessment of pancreatoduodenectomy specimens: an overview of different grossing approaches and the relevance of the histopathological characteristics in the oncologic assessment of pancreatoduodenectomy specimens. Virchows Arch.

[27] Shrikhande SV, Kleeff J, Reiser C (2007). Pancreatic resection for M1 pancreatic ductal adenocarcinoma. Ann Surg Oncol.

[28] Yamada H, Hirano S, Tanaka E (2006). Surgical treatment of liver metastases from pancreatic cancer. HPB (Oxford).

