# Supplementary material for: The diversity between curatively resected pancreatic head and body-tail cancers based on the 8th edition of AJCC staging system: a multicenter cohort study
Source: BMC Cancer. 2019 Oct 22;19:981. doi: 10.1186/s12885-019-6178-z (PMC6805668; doi:10.1186/s12885-019-6178-z)
Supplement: Supplementary file 1 — Additional file 1: Table S1. 8th AJCC stage for PC. The details of TNM Stage in 8th edition of American Joint Committee on Cancer according to primary tumor, regional lymph node and Distant metastasis. [file 12885_2019_6178_MOESM1_ESM.docx]

Supplemental Table 1 8^th^ AJCC stage for PC

| Primary tumor (T) | Regional lymph nodes (N) | | Distant metastases (M) |
| --- | --- | --- | --- |
| T1 Maximum tumor diameter ≤2 cm | N0 | No regional lymph node metastasis | M0 No distant metastasis |
| T2 Maximum tumor diameter>2 cm ≤4 cm | N1 | Metastasis in 1–3 regional lymph nodes | M1 Distant metastasis |
| T3 Maximum tumor diameter >4 cm T4 Tumor involves the celiac axis or the superior mesenteric artery   Stage  Stage IA | N2  T1 | Metastasis in ≥4 regional lymph nodes  N0 M0 |  |
| Stage IB | T2 | N0 M0 |  |
| Stage IIA | T3 | N0 M0 |  |
| Stage IIB | T1–T3 | N1 M0 |  |
| Stage III | T4 | Any N M0 |  |
|  | Any T | N2 M0 |  |
| Stage IV | Any T | Any N M1 |  |
