# Supplementary material for: The diversity between curatively resected pancreatic head and body-tail cancers based on the 8th edition of AJCC staging system: a multicenter cohort study
Source: BMC Cancer. 2019 Oct 22;19:981. doi: 10.1186/s12885-019-6178-z (PMC6805668; doi:10.1186/s12885-019-6178-z)
Supplement: Supplementary file 2 — Additional file 2: Table S2. 7th AJCC stage for PC. 7th AJCC stage for PC. The details of TNM Stage in 7th edition of American Joint Committee on Cancer according to primary tumor, regional lymph node and Distant metastasis. [file 12885_2019_6178_MOESM2_ESM.docx]

Supplemental Table 2 7^th^ AJCC stage for PC

| Primary tumor (T) | Regional lymph nodes (N) | | Distant metastases (M) |
| --- | --- | --- | --- |
| T1 Tumor limited to the pancreas, <2 cm in greatest dimension | N0 | No regional lymph node metastasis | M0 No distant metastasis |
| T2 Tumor limited to the pancreas, >2 cm in greatest dimension | N1 | Regional lymph node metastasis | M1 Distant metastasis |
| T3 Tumor extends beyond the pancreas but without involvement of the celiac axis or the superior mesenteric artery T4 Tumor involves the celiac axis or the superior mesenteric artery (unresectable primary tumor)  Stage  Stage IA | T1 | N0 M0 |  |
| Stage IB | T2 | N0 M0 |  |
| Stage IIA | T3 | N0 M0 |  |
| Stage IIB | T1–T3 | N1 M0 |  |
| Stage III | T4 | Any N M0 |  |
| Stage IV | Any T | Any N M1 |  |
